# Bridged oxide nanowire device fabrication using single step metal catalyst free thermal evaporation†

**DOI:** 10.1039/c7ra11987a

**Published:** 2018-03-14

**Authors:** Mustafa Coşkun, Matthew M. Ombaba, Fatih Dumludağ, Ahmet Altındal, M. Saif Islam

**Affiliations:** Faculty of Engineering and Natural Sciences, Department of Engineering Physics, Istanbul Medeniyet University 34700 Üskudar Istanbul Turkey; Department of Physics, Marmara University 34722 Kadıköy Istanbul Turkey; Department of Electrical and Computer Engineering, University of California 2064 Kemper Hall Davis California 95616 USA sislam@ucdavis.edu; Department of Physics, Yıldız Technical University 34220 Esenler Istanbul Turkey

## Abstract

In this study, indium-tin-zinc-oxide (ITZO) and Zn doped In_2_O_3_ nanowires were directly grown as bridged nanowires between two heavily doped silicon (Si) electrodes on an SOI wafer using single step vapor–solid–solid (VSS) growth method. SEM analysis showed highly dense and self aligned nanowire formation between the Si electrodes. Electrical and UV response measurements were performed in ambient condition. Current–voltage characteristics of devices exhibited both linear and non-linear behavior. This was the first demonstration of bridged ITZO and Zn-doped In_2_O_3_ nanowires. Our results show that bridged nanowire growth technique can be a potential candidate for high performance electronic and optoelectronic devices.

## Abstract 2

In this study, bridge metal-oxide nanowires grown directly between two Si electrodes on SOI wafer and their electrical performance were investigated.

## Graphical abstract

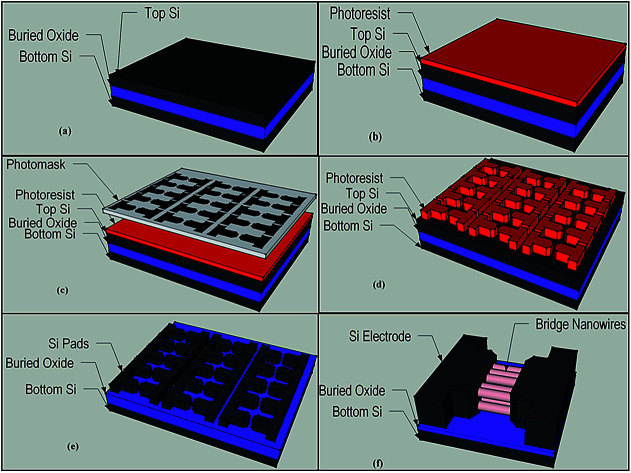

## 1. Introduction

1D semiconductor nanomaterials have generated great interest due to their unique physical and chemical properties for last few decades.^1–4^ Especially, nanowires based electronic devices such as field effect transistor (FET),^5,6^ sensor,^7–10^ photovoltaic (PV),^11^ light emitting diode,^12^ detector,^13,14^ field emitter,^15^ memory device,^16,17^ Li-ion battery^18,19^*etc.* studies showed that nanowires should be promising candidate for emerging electronic applications. High surface to volume ratio, fast charge transport and reduced grain boundary are unique properties of nanowires.^20^ Metal-oxide materials like ZnO, SnO_2_ and In_2_O_3_ are promising candidates for one dimensional nanomaterials because of their unique physical and chemical properties such as large optical band-gap, transparency, high mobility *etc.*^21–24^ So far, tremendous methods such as vapor–liquid–solid (VLS),^25^ vapor–solid–solid (VSS),^26^ template assisted,^27^ laser ablation,^28^ solution process,^29^*etc.* have been developed for the synthesis of nanostructured materials. Among them vapor–solid–solid (VSS) method is one of the best potential candidate method for growth nanowire at high temperature without using metal catalysis layer.

Nanowire based electronic device fabrication still faces some fabrication challenges including nanowire transfer from the mother substrate to the secondary substrate for device fabrication,^30^ proper alignment on the device substrate^31^ or appropriate masking and lithography enabled pattering^32^ for depositing metal electrodes. In recent years, some methods such as electric field assisted,^33^ magnetic field^34^ and micro-fluidics enabled alignment^35^ have been developed in order to align the nanowires in proper orientation on the substrate. However, these transfer and alignment methods don't eliminate the need for growing nanowires in place. Nanowire transfer processes are also tedious and time consuming. All these make the direct growth of nanowires in the shape of bridges between electrodes a highly attractive method. The process can additionally help in self-aligning the nanowires between electrodes and can simultaneously address high throughput and high performance contact formation.

In this study, we report an easy single-step thermal evaporation method to grow the bridged nanowires between two heavily doped Si electrodes on an SOI wafer without using metal catalyst or carrier gases. Structural and chemical analyses were carried out using SEM, EDX and XRD. Electrical and optical measurements were performed using heavily doped Si pads as electrodes without depositing metal on them.

## 2. Experimental

**Fig. 1:**
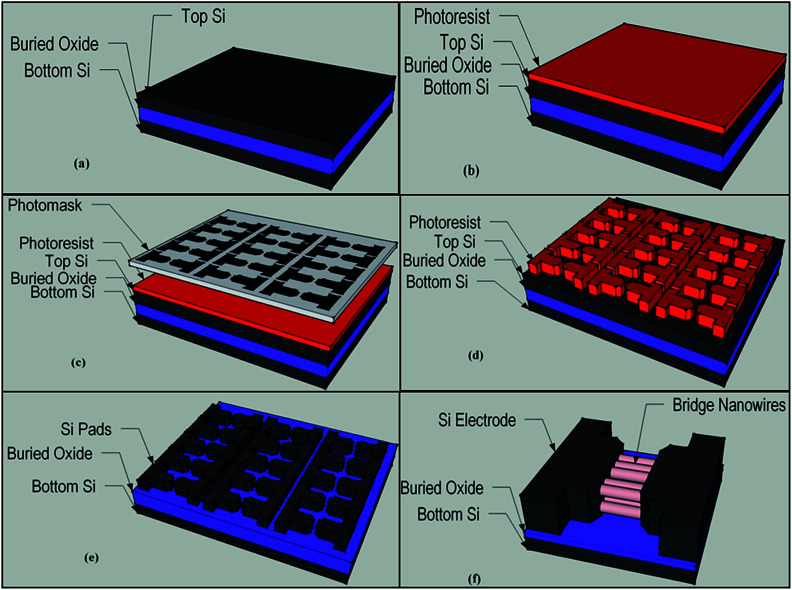
SOI wafer patterning flow diagram: (a) unprocessed SOI wafer, (b) after the deposition of photoresist, (c) photomask patterning photoresist, (d) after photolithography, (e) patterning Si device layer using hot KOH or reactive ion etching, (e) after the growth of nanowires between heavily doped Si electrodes (bridged nanowires).

ZnO (J. T. Baker), SnO_2_ (Alfa Aesar) and In_2_O_3_ (Alfa Aesar) powder and graphite ash (Alfa Aesar) mixture were used for nanowire growth between two heavily boron doped (5–8 × 10^18^ cm^−3^) p^+^ type Si (110) pads on a SOI wafer. Si pads patterning procedure is illustrated as diagrams in Fig. 1. As shown in Fig. 1, photolithography method was used to pattern Si pads on the SOI wafer. Excess Si was etched by using KOH solution or reactive ion etching and thereby Si pads were patterned on the SOI wafer. In order to grow nanowires, single zone high temperature furnace (MTI) was used. ZnO, SnO_2_ and In_2_O_3_ powder and graphite mixed in different mass ratios then were put in alumina crucible and inserted to the center of an alumina tube furnace. Pre-patterned SOI wafers and bare Si wafers that used for conduct XRD analysis, were placed close to the source material. Alumina tube was vacuum pumped (70 cm-Hg) before the nanowire growth process and during the process was kept at constant pressure of 70 cm-Hg. Alumina tube temperature was set to 1225 °C at the center and the nanowire growth process continued at constant temperature of 1225 °C for 90 minutes. Finally, after the nanowire growth process was completed, the furnace was naturally cooled down to the room temperature keeping it under the vacuum. The chemical and structural analysis of bridged nanowire were carried out using scanning electron microscopy (SEM and EDX, Hitachi S-4100), energy dispersive X-ray spectroscopy (EDX) and X-ray diffractometer (XRD, Bruker D8 Discover) analyses. Electrical characterization and UV response measurement were performed in ambient condition using Karl Suss probe station connected Agilent 4156-C semiconductor parameter analyzer and Thorlab 365 nm UV LED (M365F1).

## 3. Results and discussion

**Fig. 2:**
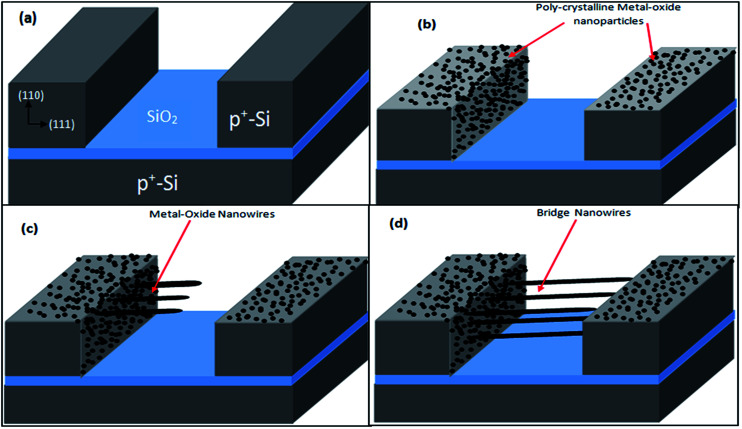
Metal-oxide nanowire growth steps: (a) heavily doped patterned SOI wafer with surface orientations before the nanowire growth process, (b) first, the powders tend to deposit on the edge of Si electrodes because of high surface energy of some sites, (c) these powders behave like a seed catalyst layer and nanowires start to grow from these site. (d) Completed growth process results in bridged nanowires between heavily doped Si electrodes.

SOI wafer pattern and bridged nanowire growth mechanism flow diagrams are illustrated in Fig. 1 and 2. The detail of lithography and etching process can be found in our previous studies.^36–38^ Before nanowire growth, patterned SOI and bare Si wafer were ultrasonically cleaned using acetone, isopropyl alcohol and de-ionized water, respectively. After cleaning procedure, SOI and bare Si wafer were placed close to the source material. In order to obtain enough nanowires formed between the Si electrodes during the growth process, a few degrees tilt was given to the SOI substrates. During the nanowire growth process, any carrier gas was not used in order to prevent high density filling of the gaps by the source material powder. Surface morphology of crystalline substrates plays an important role in the nanowire growth kinetics.^37,39^ Anisotropy in crystalline substrate surface strongly influence the growth of nanowires because of anisotropic strain that can restrict diffusion of atoms through one dimension.^39^ Nucleation is the first step for the nanowire growth and it tends to form at edges and steps of a surface because it reduce the surface energy on anisotropic surfaces.^40,41^ Thus, Si electrode edges on pre-patterned SOI substrate can provide such an appropriate site to start nucleation and consequently grow highly oriented nanowires. This situation is illustrated in Fig. 2. The source powder condenses on the Si electrode edges and form polycrystalline metal-oxide nanoparticles. Those nanoparticles start to act as seed or catalyst to grow nanowires. Subsequently, nanowires start to grow from those polycrystalline nanoparticles on (111)-oriented side-walls of the Si electrodes.

**Fig. 3:**
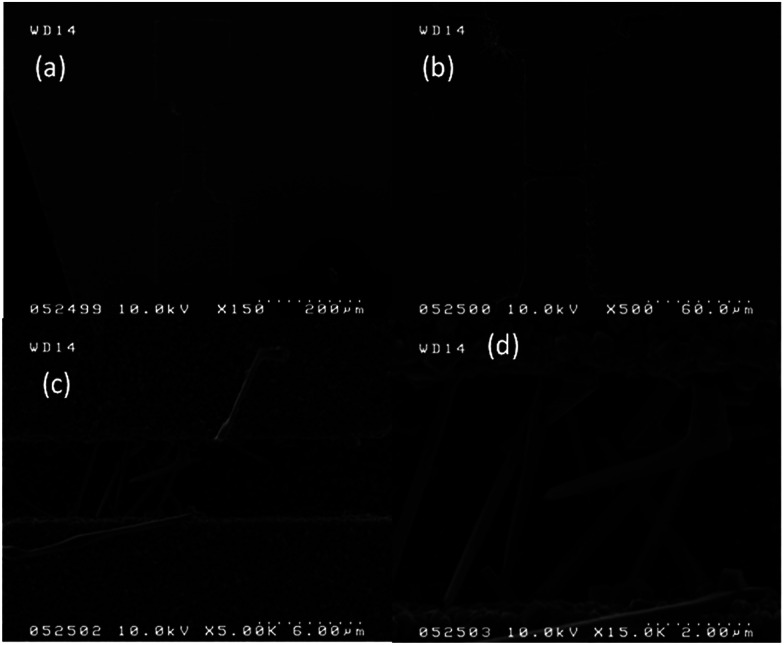
(a) Low magnification SEM image of Si electrodes on SOI wafer for first ITZO device. (b) ITZO nanowires are seen both on Si electrode sides and gap between Sii electrodes. (c) High magnification SEM image of a gap between Si electrodes on a SOI wafer. (d) High magnification SEM images of bridged ITZO nanowires that grown between Si electrodes. As seen from the SEM images, only 6 ribbon shaped nanowire are connected to two the Si electrodes.

We broke the nanowires between two electrodes and found not conductivity (see Fig. S12†). This proves that there is no thin film between the Si electrodes on SOI wafer. Fig. 3 shows SEM images of ITZO nanowires (first ITZO device) prepared at 600 °C for 90 min. Without using metal catalyst and carrier gas. EDX analysis confirmed chemical composition of ITZO based on the peaks associated with In, Sn, Zn and O atoms. The gap between two Si electrodes and the thickness of Si electrodes was 3 μm and 2 μm, respectively. As seen in Fig. 3, the nanowires exhibit ribbon-like shape with a rectangular cross-section area of 60 nm × 230 nm. The illustration of the nanowire shape and sizes are presented in Fig. 5a. As mentioned before anisotropic surfaces like Si electrode walls' edges serve as the nucleation sites and nanowires grow in highly dense formation, as shown in Fig. 3. Current–voltage (*I*–*V*) characteristic of bridged ITZO nanowire conducted under dark ambient condition and result is given in Fig. 6. Nearly linear *I*–*V* behavior was obtained from the electrical measurements and the device showed high conductivity.

**Fig. 4:**
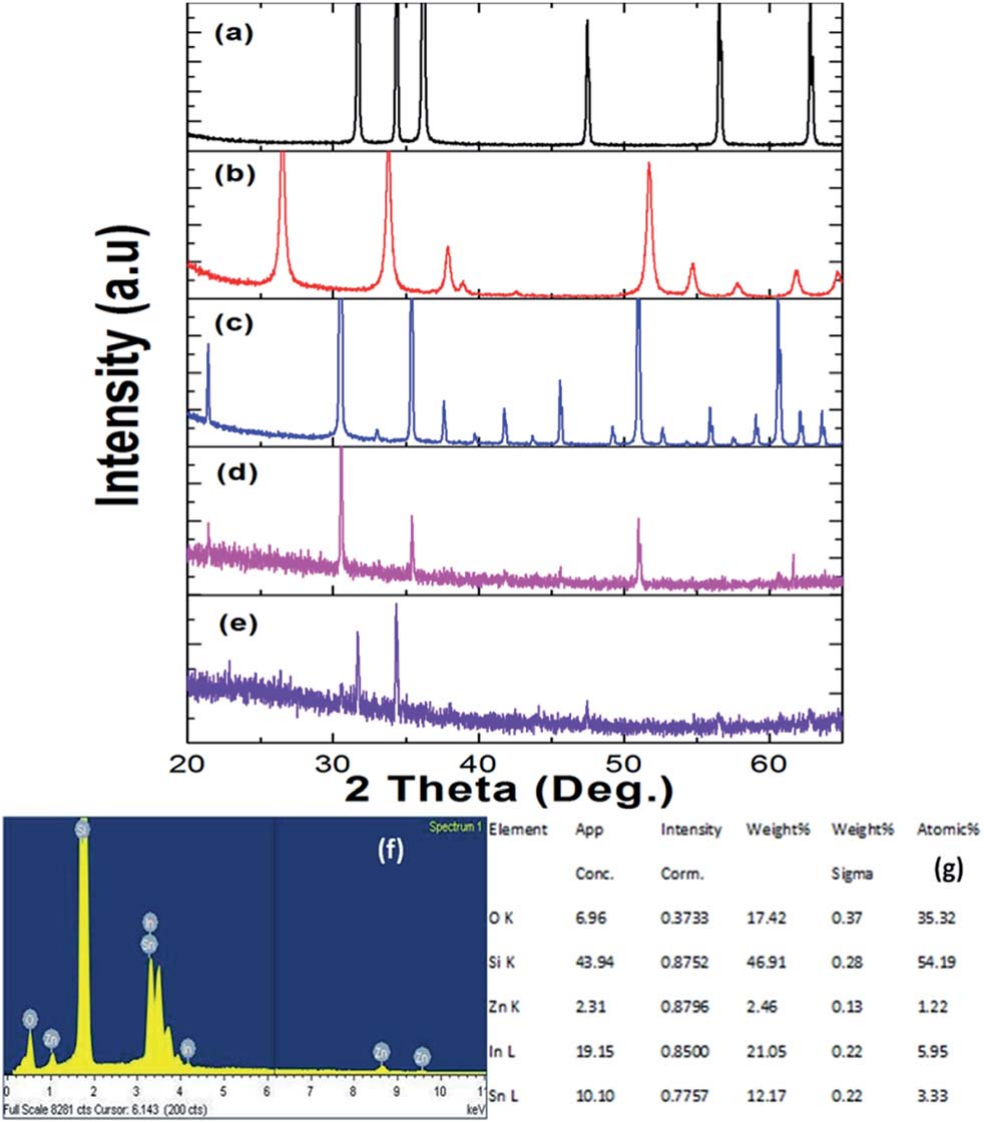
XRD analysis result of (a) ZnO (PDF 01-089-1397), (b) SnO_2_ (PDF 01-070-4175), (c) In_2_O_3_ (PDF 00-044-1087), (d) the first bridged ITZO nanowire device, (e) the second bridged ITZO Nanowire device, (f and g) EDX analysis result of first ITZO nanowire.

**Fig. 5:**
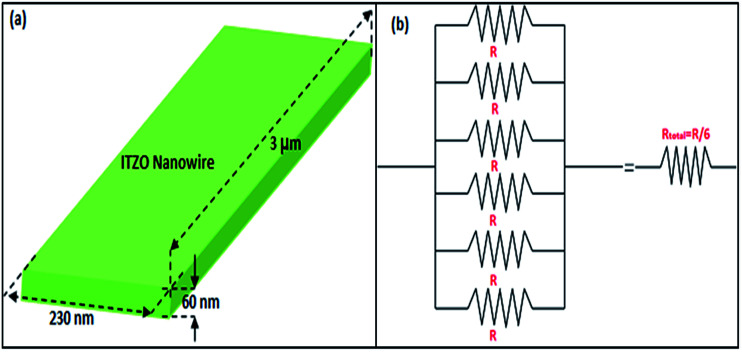
(a) Illustration of ribbon shaped nanowire calculated using SEM images. (b) Equivalent circuit of the bridged ITZO nanowire device.

**Fig. 6:**
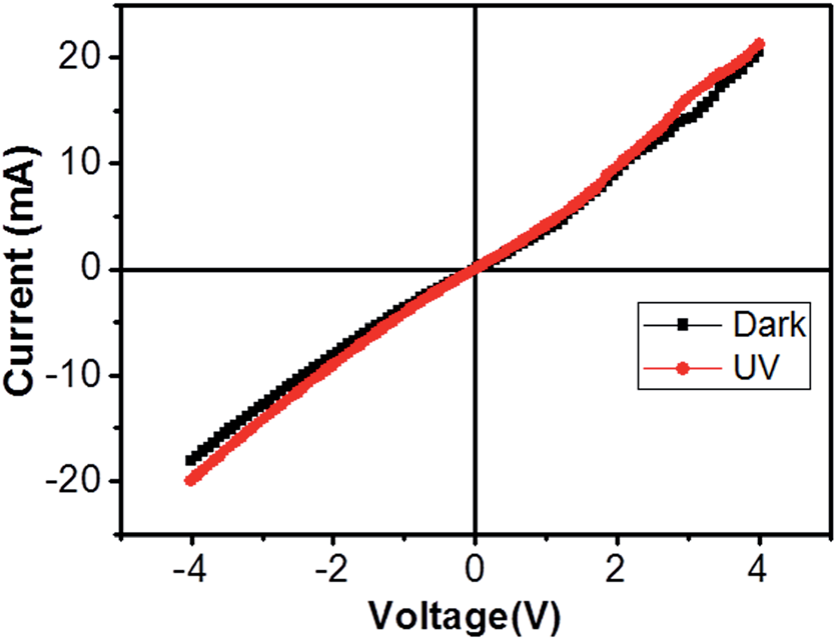
Current–voltage characteristic of bridged ITZO nanowire under dark and UV (365 nm) light. The device showed very little response to UV light because of its high conductivity.

ITZO nanowire XRD and EDX analysis results are presented in Fig. 4. We also present the XRD data of the source powders (ZnO, SnO_2_ and In_2_O_3_) with PDF card number (see Fig. 4a–c). As can be seen from the Fig. 4. First and second ITZO nanowire show predominant In_2_O_3_ and ZnO phases. Such observation has been reported on ITZO thin films in literature by Ni and co workers.^42^ According to that study, ITZO thin film composition depends on elemental ratio and strongly influences the XRD peaks. The study clearly showed that the high In content in ITZO film resulted dominant In_2_O_3_ phase in the XRD analysis. When Ni *et al.* decreased the In content and increased Zn and Sn ratios, they observed ZnO and/or SnO_2_ phase in XRD patterns. Our results are completely in agreement with this study.^42^ As can be seen from the Fig. 4(d) and (e), first device with ITZO nanowire shows In_2_O_3_ phase while second device shows both In_2_O_3_ and ZnO phases due to the high In and Zn content. As is reported in literature, increasing ZnO ratio in the SnO_2_ and In_2_O_3_ metal-oxide materials results lower conductivity.^43^

From EDX analysis (see Fig. 4), we can clearly see that Sn and In ratio are much more higher than the Zn ratio. Hence, the lower Zn ratio in the nanowire provides higher conductivity. Current–voltage characteristics is almost linear showing mA range current at low bias voltages (Fig. 6). This result show us the bridged ITZO nanowires and their ITO like characteristics because of high In, Sn and low Zn atomic ratio. The linear *I*–*V* characteristic of the ITZO nanowire based device reveals that the energy barrier between p^+^-Si and ITZO nanowires is low. The measured resistance *R*_total_ was 222.2 Ω. As shown in Fig. 3, the device has only 6 nanowire between two Si electrodes and they are almost parallel. If we assume that all the nanowires have same resistance value, the total resistance should be equal *R*_total_ = *R*/6 = 222.2 Ω. Thus, individual nanowire resistance is 1333.2 Ω. The resistivity (*ρ*) can be calculated using eqn (1):1
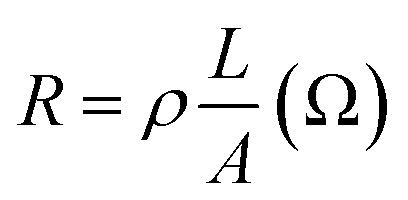
where *R*, *ρ*, *L* and *A* are the resistance, resistivity, length and cross-sectional area of an individual nanowire. Resistivity of one nanowire was calculated as *ρ* = 6.13 × 10^−4^ Ω cm which is comparable with reported values in literature.^44^ Conductance (*G*) and conductivity (*σ*) values were calculated using eqn (2) and (3):2
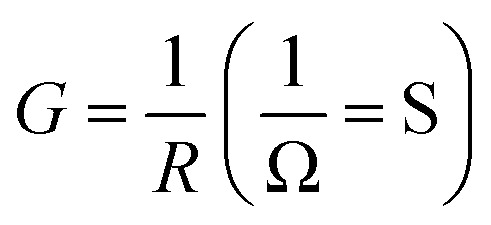
3
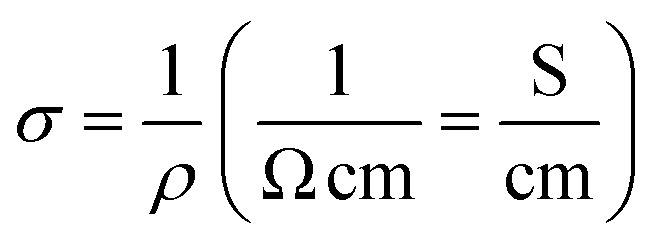


Total conductance and conductance of individual nanowire were calculated by using the *I*–*V* curves as *G*_total_ = 4.5 × 10^−3^ (s) and *G*_total_ = 7.5 × 10^−4^ (s). Conductivity of individual nanowire was calculated as *σ* = 1.63 × 10^−3^ (S cm^−1^). It was observed that the measured conductivity value of the ITZO nanowire is higher than the reported values of ITZO thin film.^45^

**Fig. 7:**
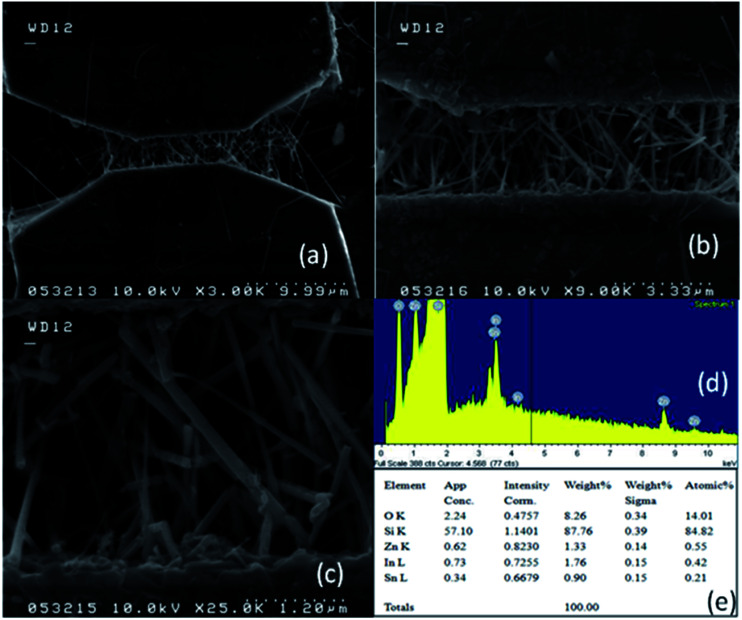
(a) Low magnification SEM image of Si electrodes and ITZO nanowires between them on a SOI wafer for second ITZO device. (b) Medium and, (c) high magnification SEM images of ITZO nanowires between Si electrodes. (d and e) EDX analysis of bridged ITZO nanowire for the second category of devices.

**Fig. 8:**
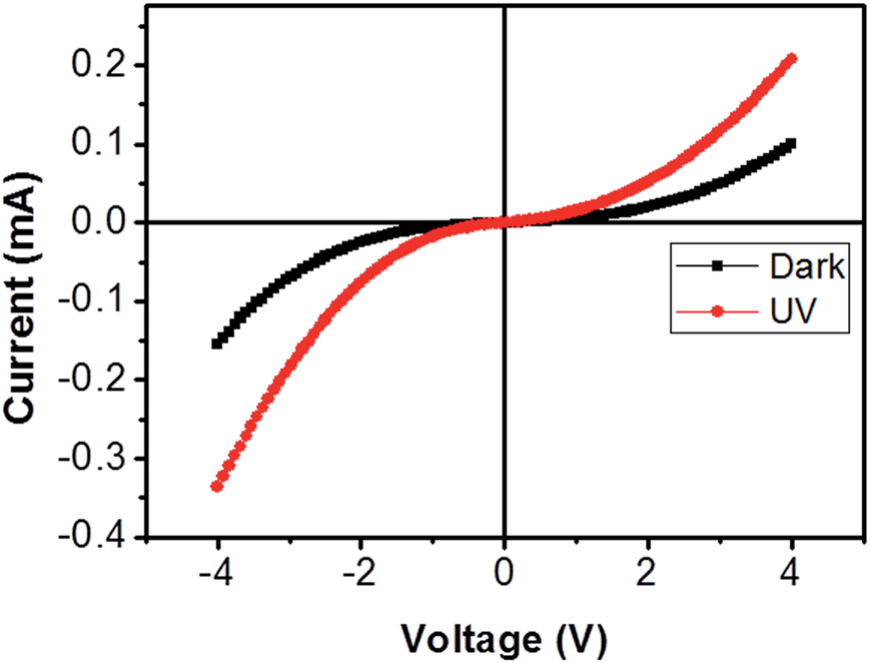
Current–voltage curves of second ITZO nanowire device in dark and under UV light.

A number of bridged ITZO nanowires SEM images are presented for second ITZO device in Fig. 7. As seen in the figure, highly dense nanowires are formed between Si electrodes on (111) orientated side-walls. Average nanowire diameter and the gap between Si electrodes were calculated roughly as 125 nm and 3 μm, respectively. EDX analysis confirmed chemical composition of ITZO material based on the peaks associated with In, Sn, Zn and O atoms (Fig. 7d and e). XRD analysis is presented in Fig. 4e. Estimating the number of nanowires between the Si electrodes is highly difficult because of highly dense nanowire formation. Hence, it is challenging to estimate the electrical parameter, unlike the aforementioned case. Current–voltage characteristic of the device deviate from linearity and the device showed non-linear behavior (see Fig. 8). Such a behavior was observed reported earlier for ZnO nanowires.^37^ This can be attributed an energy barrier formation between Si electrodes and ITZO nanowire. Also if we compare to the previously mentioned ITZO device (first ITZO device), we can clearly see that this device exhibit lower conductivity and current value. This clearly originates from the composition of the nanowires that have low In and Sn ratio with relatively high Zn ratio. Increasing Zn ratio causes decreasing conductivity. Changing chemical composition directly affect some electrical and optical parameters such as conductivity, energy barrier and band gap.

We can see this clearly from the *I*–*V* measurements of the first and second ITZO nanowire devices. Measurements of UV (365 nm) response revealed that the device did not show adequate response to UV light. We assume that there are two possible reasons that can explain this low UV response: first, UV light cannot reach to all of the nanowires because the nanowires in the upper end hinder the UV light in reaching the bottom end. Second, nanowires may have large band-gap to remain transparent under UV light of 365 nm wavelengths.

**Fig. 9:**
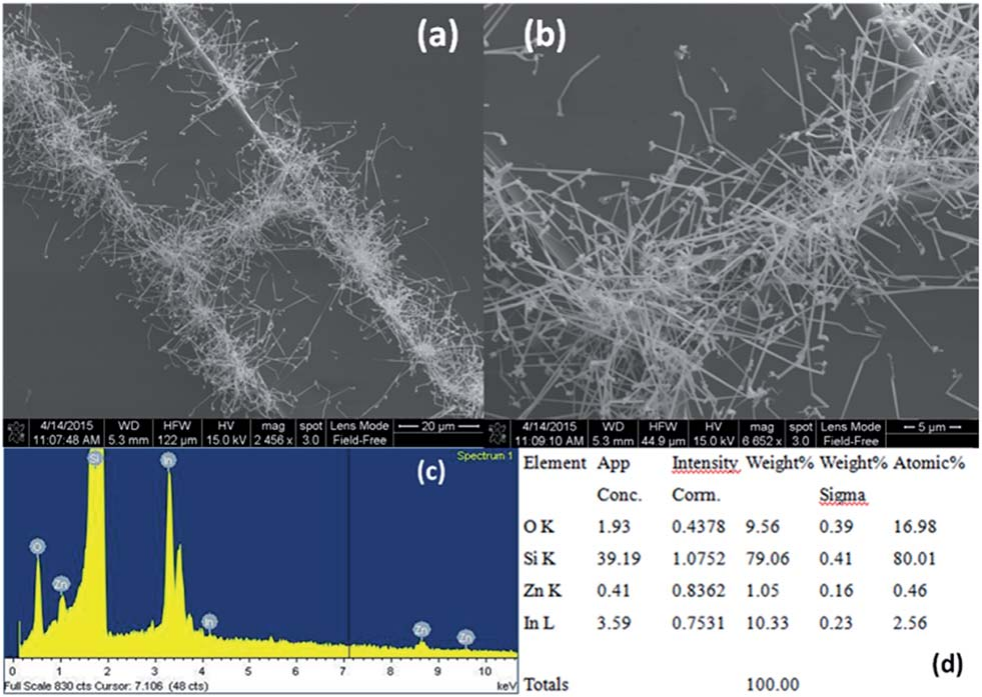
(a) Low and (b) high magnification SEM images of bridge Zn doped In_2_O_3_ nanowire devices. Clearly seen from the images that high dense nanowires grown on the vertical walls of Si electrode' and not on the planar surface. (c) and (d) show EDX analysis results of Zn doped In_2_O_3_.

**Fig. 10:**
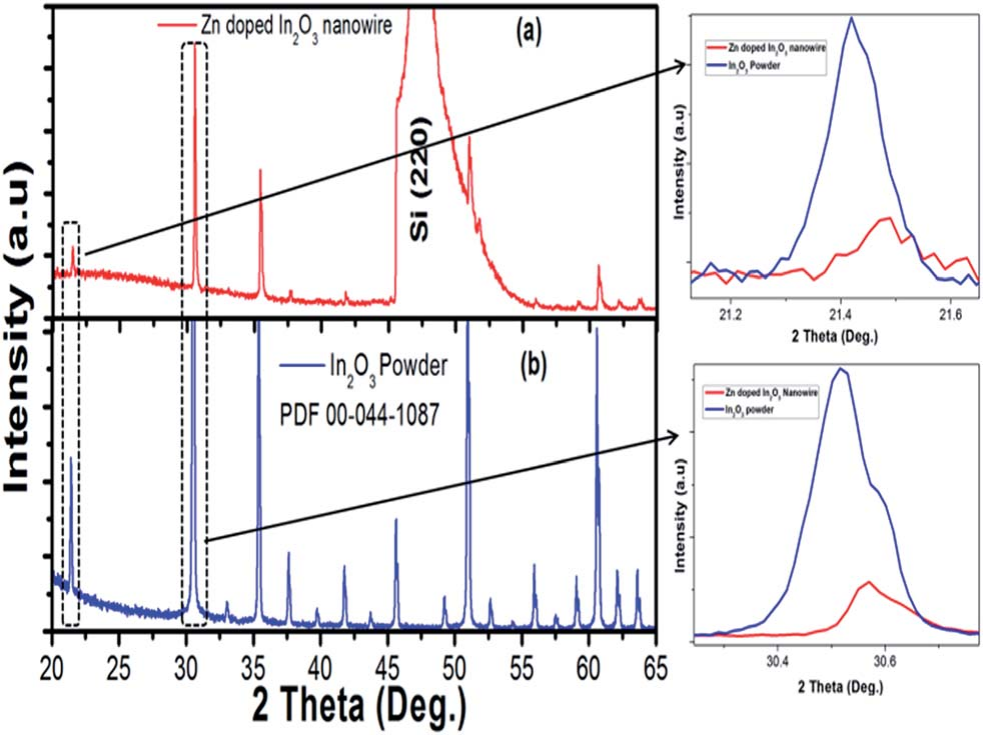
XRD analysis result of (a) In_2_O_3_ (PDF 00-044-1087), (b) bridged Zn doped In_2_O_3_ nanowire. Arrows show the peaks shift through the higher two theta values.

**Fig. 11:**
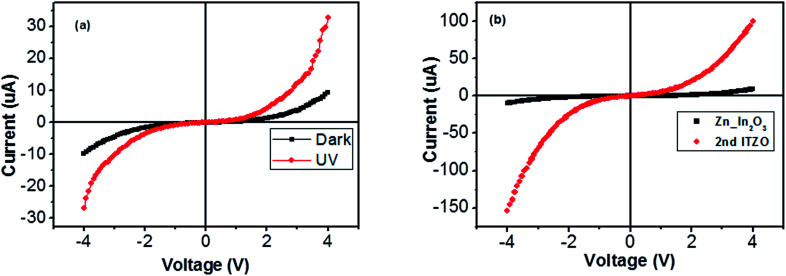
(a) Current–voltage characteristic of bridged Zn doped In_2_O_3_ nanowire device in dark and under UV light (365 nm). (b) Comparison of current–voltage characteristic of second bridged ITZO and Zn doped In_2_O_3_ devices.

Nanowires in the third device with bridged nanowires are Zn doped In_2_O_3_ nanowires. Their SEM images are given in Fig. 9. Unlike the ITZO devices, Zn doped In_2_O_3_ nanowires have sharp tips because of high ratio of indium (In).^46^ The gaps between Si electrodes are completely filled by nanowire. It can be clearly seen that almost all nanowires grow on the vertical sidewall of the Si electrodes and none of them grows on the Si surface. This indicates that the anisotropy has a strong effect on the nanowire growth because of high surface energy. XRD and EDX analyses confirmed chemical composition of Zn doped In_2_O_3_ nanowire by the atomic composition (Fig. 9c and d). XRD analyses of source powders and nanowires were carried out and results are presented in Fig. 10. As can be seen from the XRD results Zn doped In_2_O_3_ nanowire peaks slightly shifted towards higher 2 theta angles (arrows show first two peaks) as compared with that of un-doped In_2_O_3_, which confirms that Zn ions occupy In sites within In_2_O_3_ crystal lattice.^47^ This is because of different ionic radius of Zn^+2^ (0.074 nm) and In^+3^ (0.081 nm). Substituting Zn^+2^ ions into the In^+3^ site in the In_2_O_3_ resulted contraction of the lattice parameter hence peaks shifted through the higher theta values. Similar results were reported by other researchers.^48^ UV response measurement revealed (Fig. 11a) that the device has almost 3 times higher current value than the dark one. Also, the current–voltage characteristic showed that Zn doped In_2_O_3_ nanowires have lower current value than the second ITZO device (Fig. 11b). This can be attributed to the absence of Sn atoms. Doping Sn atoms into the Zn doped In_2_O_3_ nanowires will increase conductivity and materials will behave in a manner similar to the first or second ITZO devices or ITO. Hence, the results clearly show UV response result is nearly similar to the second ITZO device as we mentioned before about the possible low UV response of second ITZO device.

Other ITZO devices were presented in supporting information† section. Current–voltage characteristics, SEM and EDX results were listed for each devices, respectively. Dense nanowire formation was observed on the (111) direction of Si electrodes. Current–voltage characteristic were shown for all ITZO devices (see Fig. S11†) and it can be seen that *I*–*V* behavior of devices is almost similar with varying magnitude of currents in different devices. This is clearly related to In, Sn and Zn atomic ratio and the number of nanowire. High In and Sn atomic ratio showed that nanowires have high conductivity but high Zn ratio decreases nanowire conductivity. Also, UV light improved the nanowire conductivity due to the excited charge carriers from the valance band to conduction band^49–51^ and we observed that this improvement in the conductivity highly depends on both nanowire composition and density.

## 4. Conclusions

In this study we proposed an easy and inexpensive method to fabricate 3D bridged nanowires directly grown between two Si electrodes. SEM images show that anisotropic surfaces highly affect the nanowire growth kinetics and nanowires tend to grow on surfaces with higher energy. We observed that direct nanowire grown between isolated electrodes provide some advantages for faster device fabrication and characterization. Electrical measurements showed both linear and non-linear (like diode) current–voltage characteristics because of the energy barrier between the nanowire and Si electrodes. Our results on bridged nanowire growth technique can enable scalable manufacturing of a number of high performance devices.

## Conflicts of interest

There are no conflicts to declare.

## Supplementary Material

RA-008-C7RA11987A-s001
