# Supplementary material for: Bridged oxide nanowire device fabrication using single step metal catalyst free thermal evaporation
Source: RSC Adv. 2018 Mar 14;8(19):10294–301. doi: 10.1039/c7ra11987a (PMC9078917; doi:10.1039/c7ra11987a)
Supplement: RA-008-C7RA11987A-s001 [file RA-008-C7RA11987A-s001.pdf]

Electronic Supplementary Material (ESI) for RSC Advances.
This journal is © The Royal Society of Chemistry 2018

Electronic Supplementary Information

Besides the first, second ITZO and Zn doped In_2O_3 devices, other ITZO devices are listed in below. These devices were fabricated and electrical measurement were performed in same condition or ambient with previous ones. SEM, EDX and current-voltage characteristics are listed for all these devices.

- **Third ITZO Device**

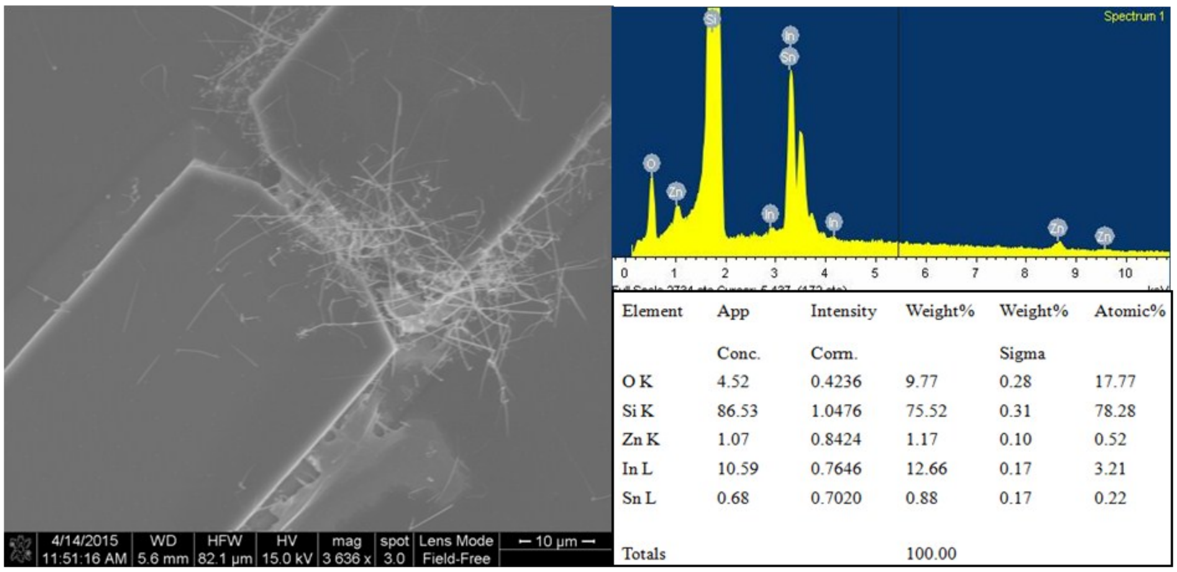

Element	App Conc.	Intensity Corrn.	Weight%	Weight% Sigma	Atomic%
O K	4.52	0.4236	9.77	0.28	17.77
Si K	86.53	1.0476	75.52	0.31	78.28
Zn K	1.07	0.8424	1.17	0.10	0.52
In L	10.59	0.7646	12.66	0.17	3.21
Sn L	0.68	0.7020	0.88	0.17	0.22
Totals			100.00		

Fig. S1. SEM and EDX analysis results of third ITZO device

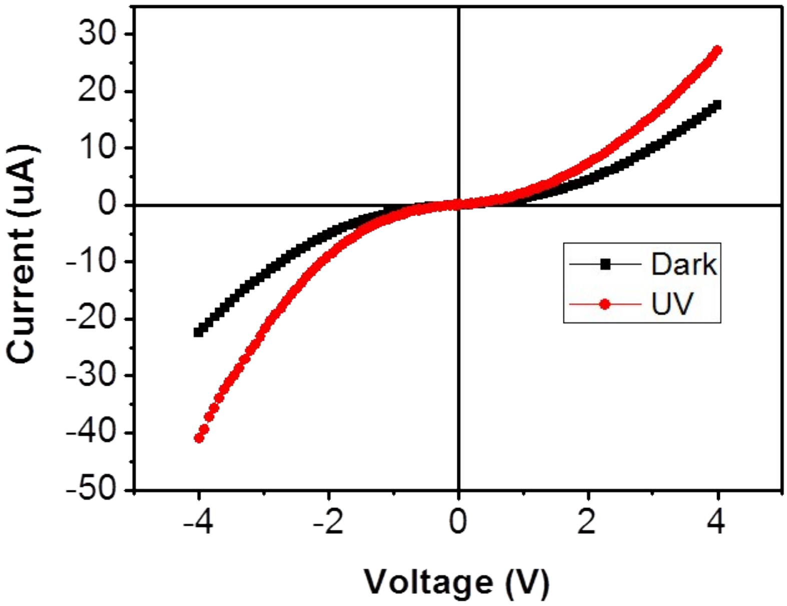

Fig. S2 I-V characteristics in dark and under UV light of third ITZO device.

- **Fourth ITZO Device**

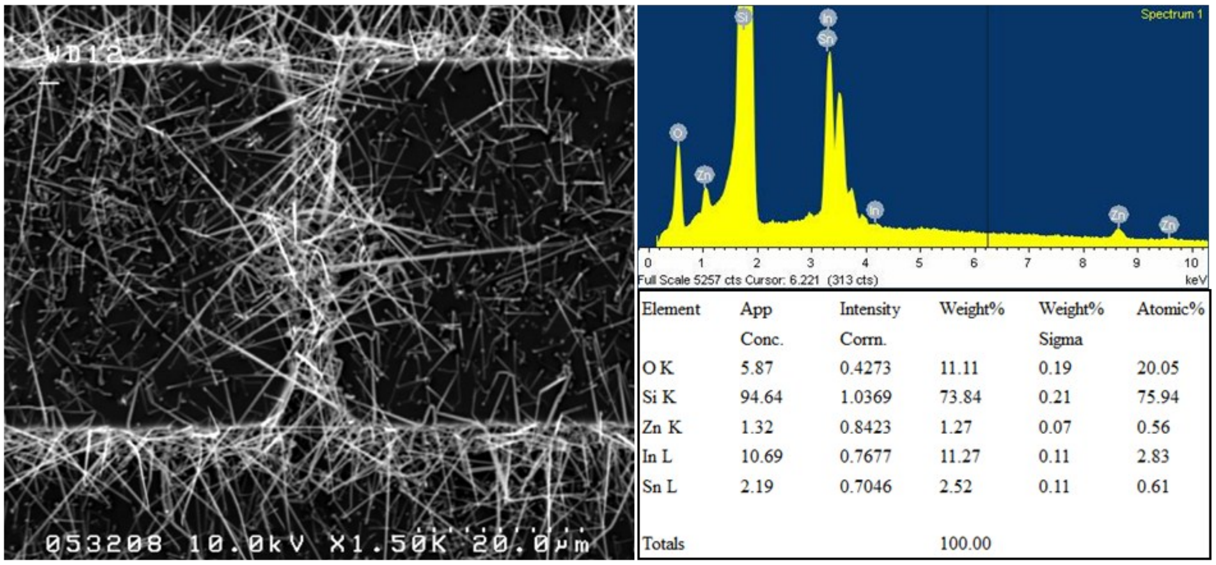

Element	App Conc.	Intensity Corrn.	Weight%	Weight% Sigma	Atomic%
O K	5.87	0.4273	11.11	0.19	20.05
Si K	94.64	1.0369	73.84	0.21	75.94
Zn K	1.32	0.8423	1.27	0.07	0.56
In L	10.69	0.7677	11.27	0.11	2.83
Sn L	2.19	0.7046	2.52	0.11	0.61
Totals			100.00		

Fig. S3 SEM and EDX analysis for the fourth ITZO device.

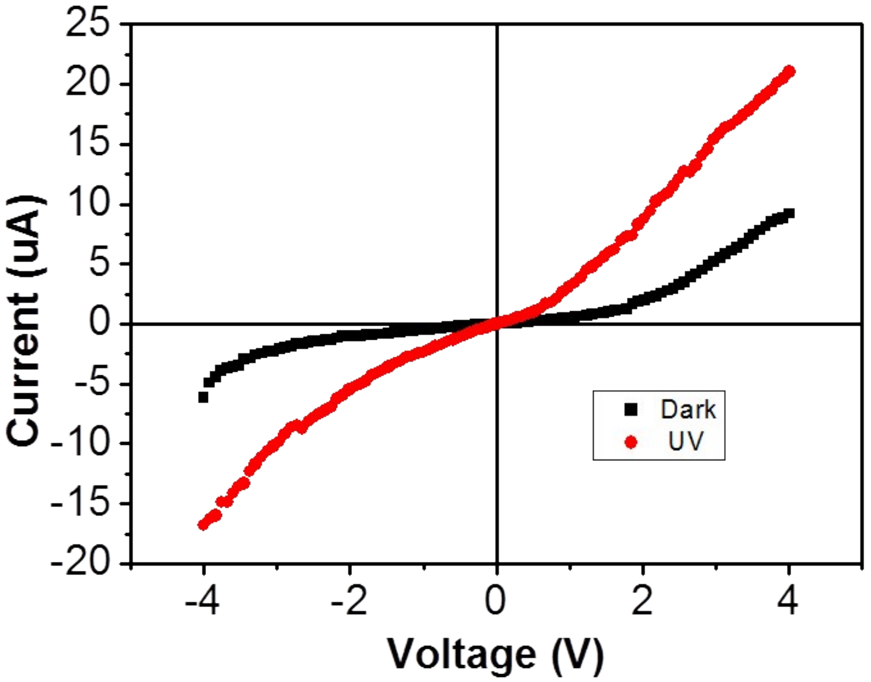

Fig. S4 Current-voltage characteristic of the fourth ITZO device in dark and under UV light.

- **Fifth ITZO Device**

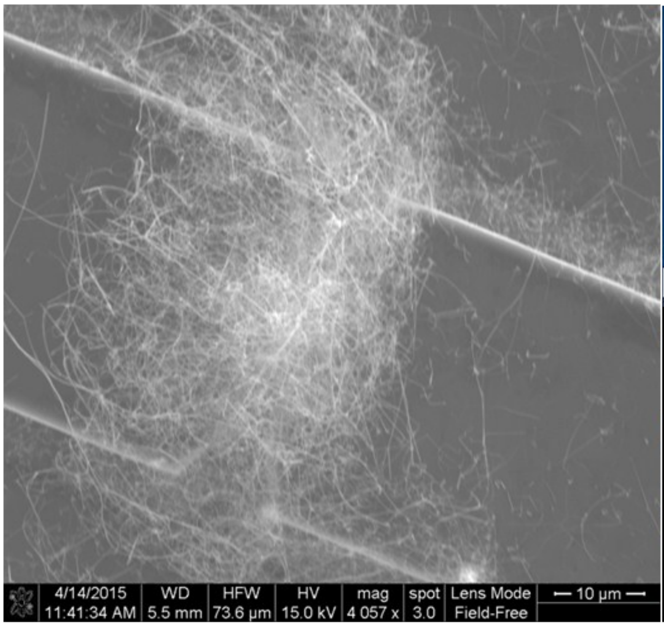

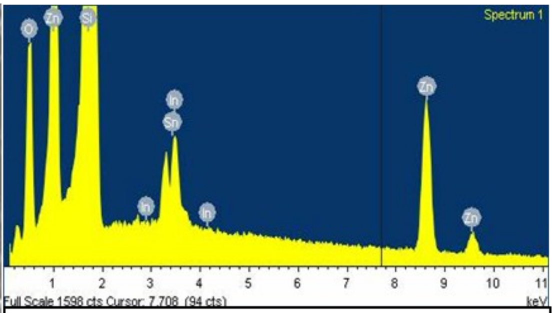

Element	App Conc.	Intensity Corrn.	Weight%	Weight% Sigma	Atomic%
O K	7.71	0.5172	11.87	0.24	21.00
Si K	86.45	0.9507	72.46	0.26	73.00
Zn K	12.16	0.8394	11.54	0.16	4.99
In L	2.72	0.7537	2.87	0.11	0.71
Sn L	1.10	0.6926	1.26	0.11	0.30
Totals			100.00		

Fig. S5 SEM and EDX analysis results of fifth ITZO device.

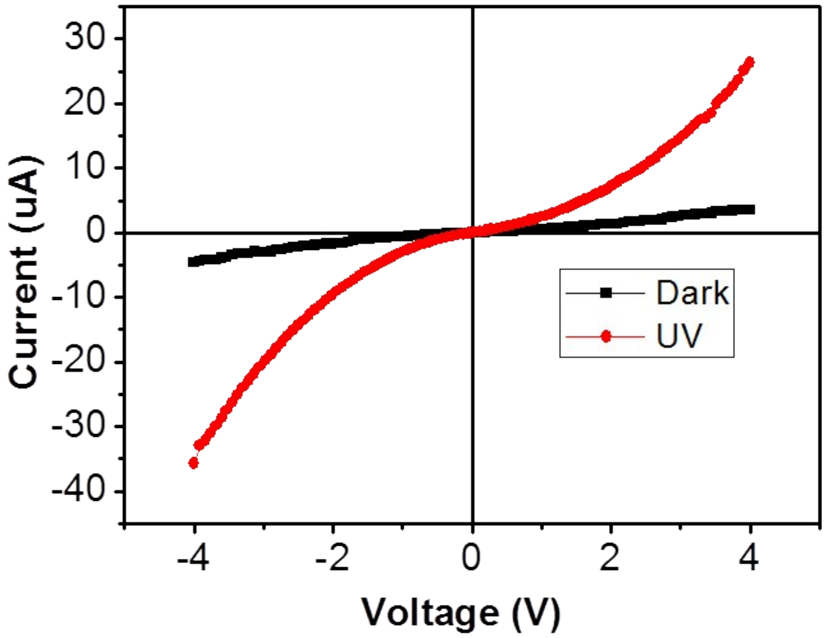

Fig. S6 Current-voltage characteristic of fifth ITZO device in dark and under UV light.

- **Sixth ITZO Device**

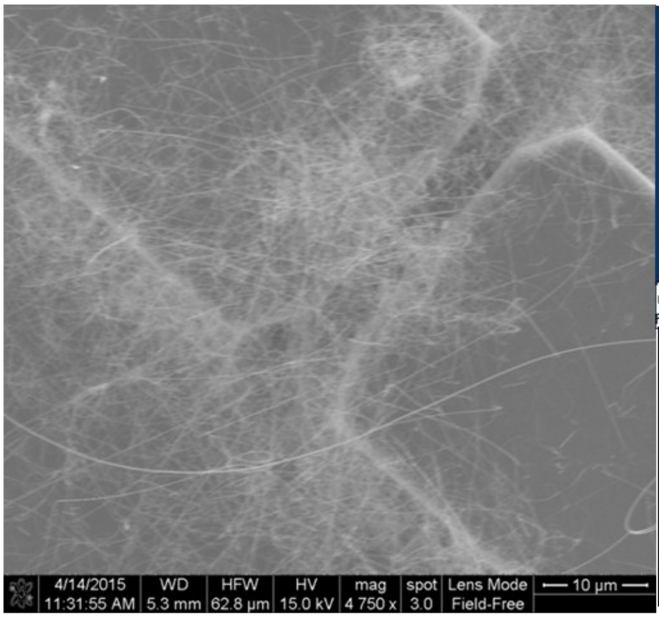

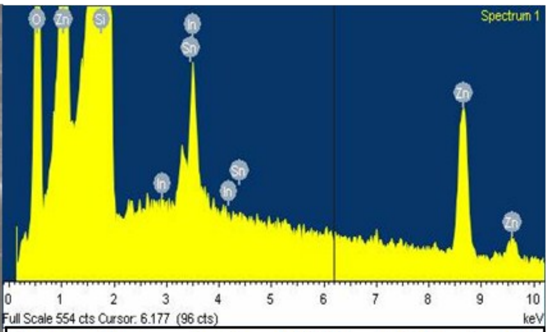

Element	App Conc.	Intensity Corrn.	Weight%	Weight% Sigma	Atomic%
O K	4.55	0.5039	10.96	0.29	18.70
Si K	69.45	1.0466	80.43	0.33	78.19
Zn K	4.07	0.8284	5.96	0.16	2.49
In L	0.86	0.7376	1.42	0.12	0.34
Sn L	0.69	0.6783	1.23	0.13	0.28
Totals			100.00		

Fig. S7 SEM and EDX analysis results of sixth ITZO device.

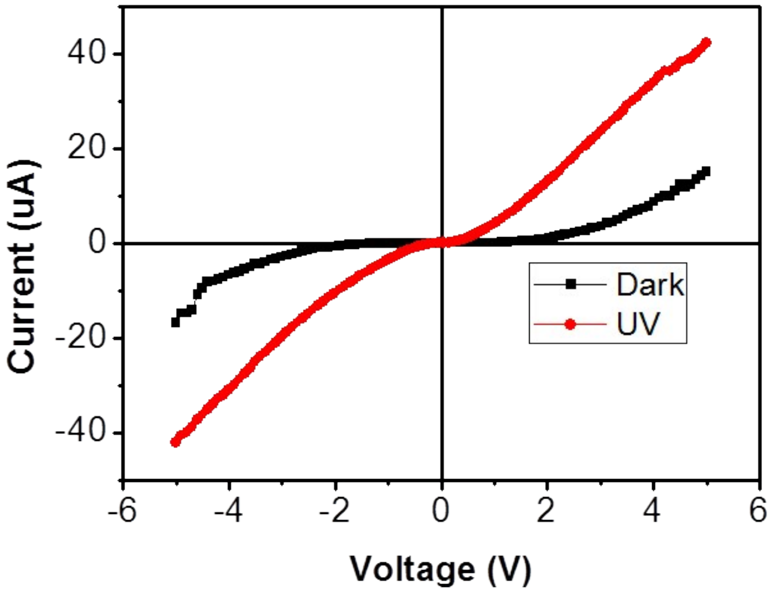

Fig. S8 Current-voltage characteristics of sixth ITZO device in dark and under UV light.

- **Seventh ITZO Device**

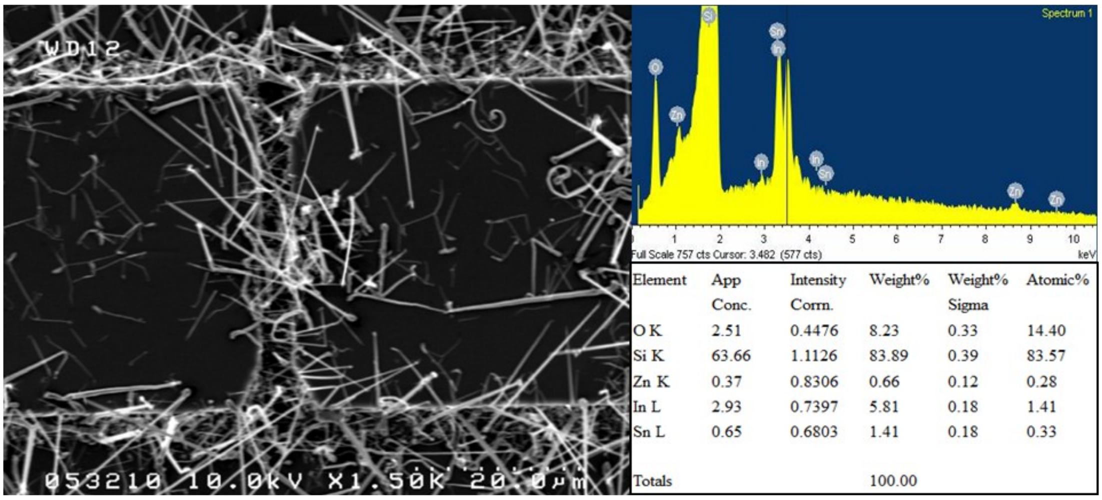

Element	App Conc.	Intensity Corrn.	Weight%	Weight% Sigma	Atomic%
O K	2.51	0.4476	8.23	0.33	14.40
Si K	63.66	1.1126	83.89	0.39	83.57
Zn K	0.37	0.8306	0.66	0.12	0.28
In L	2.93	0.7397	5.81	0.18	1.41
Sn L	0.65	0.6803	1.41	0.18	0.33
Totals			100.00		

Fig. S9 SEM and EDX analysis results of seventh ITZO device.

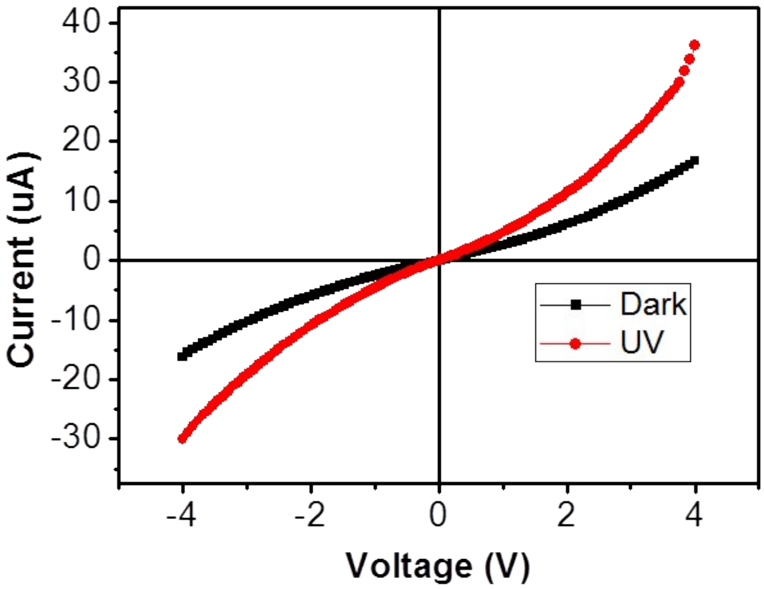

Fig. S10 Current-voltage characteristics of seventh ITZO device in dark and under UV light.

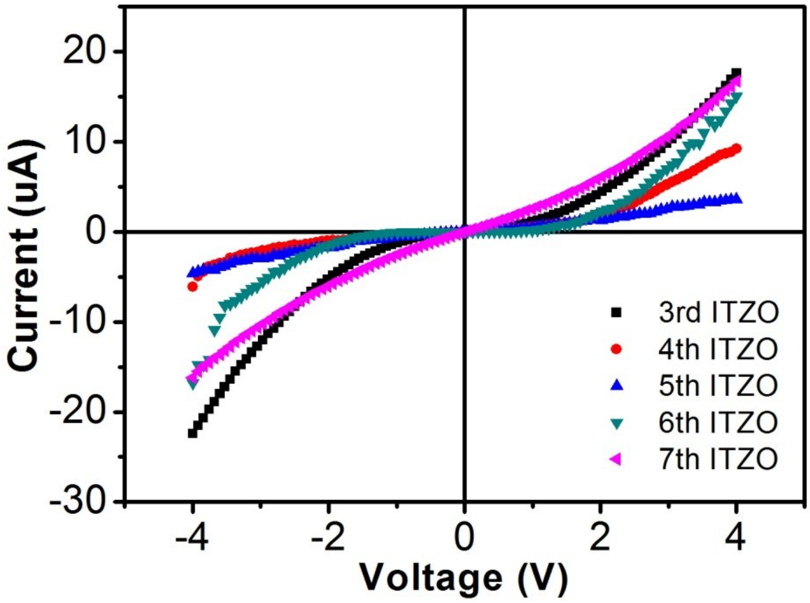

Fig. S11 Current-voltage characteristics of third, fourth, fifth, sixth and seventh ITZO device in dark.

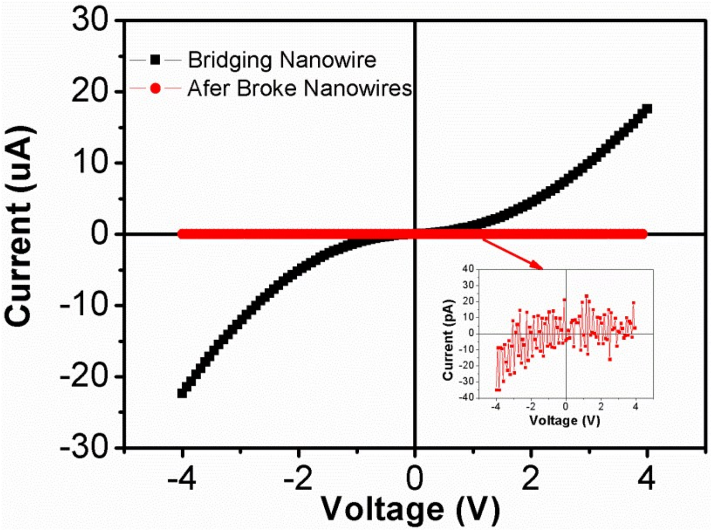

Fig. S12. The I-V measurement result of the ITZO device with nanowire and without nanowire between Si electrodes. The measurement result confirmed that no conductivity after broke the nanowires.
